# Crystal structure and Hirshfeld surface of a penta­amine­copper(II) complex with urea and chloride

**DOI:** 10.1107/S2056989024004298

**Published:** 2024-05-14

**Authors:** Olivia D. Breen, Tony D. Keene

**Affiliations:** a School of Chemistry, University College Dublin, Belfield, Dublin 4, D04 V1W8, Ireland; Universidad de la República, Uruguay

**Keywords:** crystal structure, Hirshfeld surface analysis, copper complex, deep eutectic solvent

## Abstract

The reaction of copper(II) oxalate and hexa­methyl­ene­tetra­mine in a deep eutectic solvent made of urea and choline chloride produced crystals of penta­amine­copper(II) dichloride–urea (1/1), [Cu(NH_3_)_5_]Cl_2_·CO(NH_2_)_2_, which was characterized by single-crystal X-ray diffraction. The complex contains discrete penta­amine­copper(II) units in a square-based pyramidal geometry. The overall structure of the multi-component crystal is dictated by hydrogen bonding between urea mol­ecules and amine H atoms with chloride anions.

## Abstract 2

The title multi-component crystal, [Cu(NH_3_)_5_]Cl_2_·CO(NH_2_)_2_, was synthesized from a deep eutectic solvent to yield an unusual penta­amine­copper(II) complex. Hydrogen bonding takes place between chloride ions and both the penta­amine­copper ions and urea mol­ecules.

## 1. Chemical context

Copper oxalate, Cu(ox), is primarily a synthetic compound that has been the subject of much research and can also be found naturally as the mineral moolooite (Clarke & Williams, 1986 ▸). It has been examined as a potential precursor to forming copper oxide particles with controlled morphologies (Rahimi-Nasrabadi *et al.*, 2013 ▸) and has been the subject of thorough investigation of its structure (Fichtner-Schmittler, 1984 ▸; O’Connor *et al.*, 2019 ▸; Kornyakov *et al.*, 2023 ▸). Unlike other first row transition-metal oxalate compounds, which form mainly as dihydrates, copper oxalate forms as anhydrous chains with chemisorbed water on the particle’s surface, with the amount of water being dependent on the reaction conditions. The amount of water present also contributes to disorder (O’Connor *et al.*, 2019 ▸; Kornyakov *et al.*, 2023 ▸). Copper oxalate forms as a microcrystalline powder so we were inter­ested in investigating the use of alternative solvents that could allow for the synthesis of single crystals of anhydrous copper oxalate compounds.

Ionic liquids have been used to tune reaction conditions such as solubility and have also been utilized in the synthesis of coordination compounds to help with templating of the structure (Dybtsev *et al.*, 2004 ▸). However, cost can be a prohibitive factor in their use and so deep eutectic solvents, mixtures that have melting points drastically lower than the individual components, can be a cheaper alternative (Zhang *et al.*, 2009 ▸). Choline chloride and urea are commonly used as a deep eutectic solvent due to the melting point of 285 K for the eutectic mixture, which is considerably lower than the components’ own, with melting points of 575 and 406 K, respectively (Abbott *et al.*, 2003 ▸). In the presence of hexa­methyl­ene­tetra­mine the solvent allows copper oxalate to be dissolved when heated. With this in mind, the investigation of copper compounds that could form from deep eutectic solvents was carried out with the intent of forming crystalline anhydrous copper oxalate compounds. In this particular case, while this did not occur, we have obtained a copper(II) complex with ammonia ligands, urea and chloride, [Cu(NH_3_)_5_]Cl_2_·CO(NH_2_)_2_. 
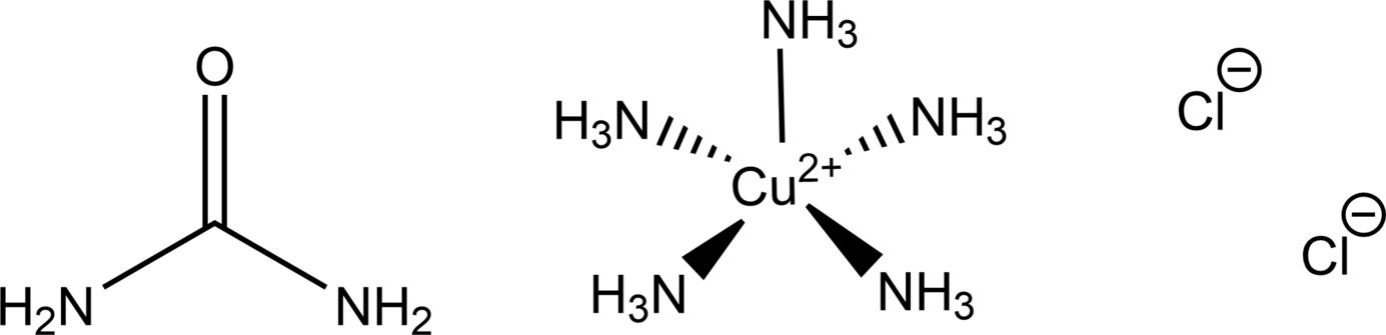


The formation of this copper(II) complex is thought to occur through the decomposition of urea to release ammonia, which is the source of the ligands. Penta­amine­copper(II) complexes are known to form when excess ammonia is present (Cotton & Wilkinson, 1972 ▸). Ammonia out-competes choline, chloride and urea as a ligand for copper(II), resulting in the penta­amine­copper(II) complex. Despite this, penta­amine­copper(II) complexes rarely crystallize, likely due to the volatility of the ammonia ligand.

## 2. Structural commentary

**Figure 1:**
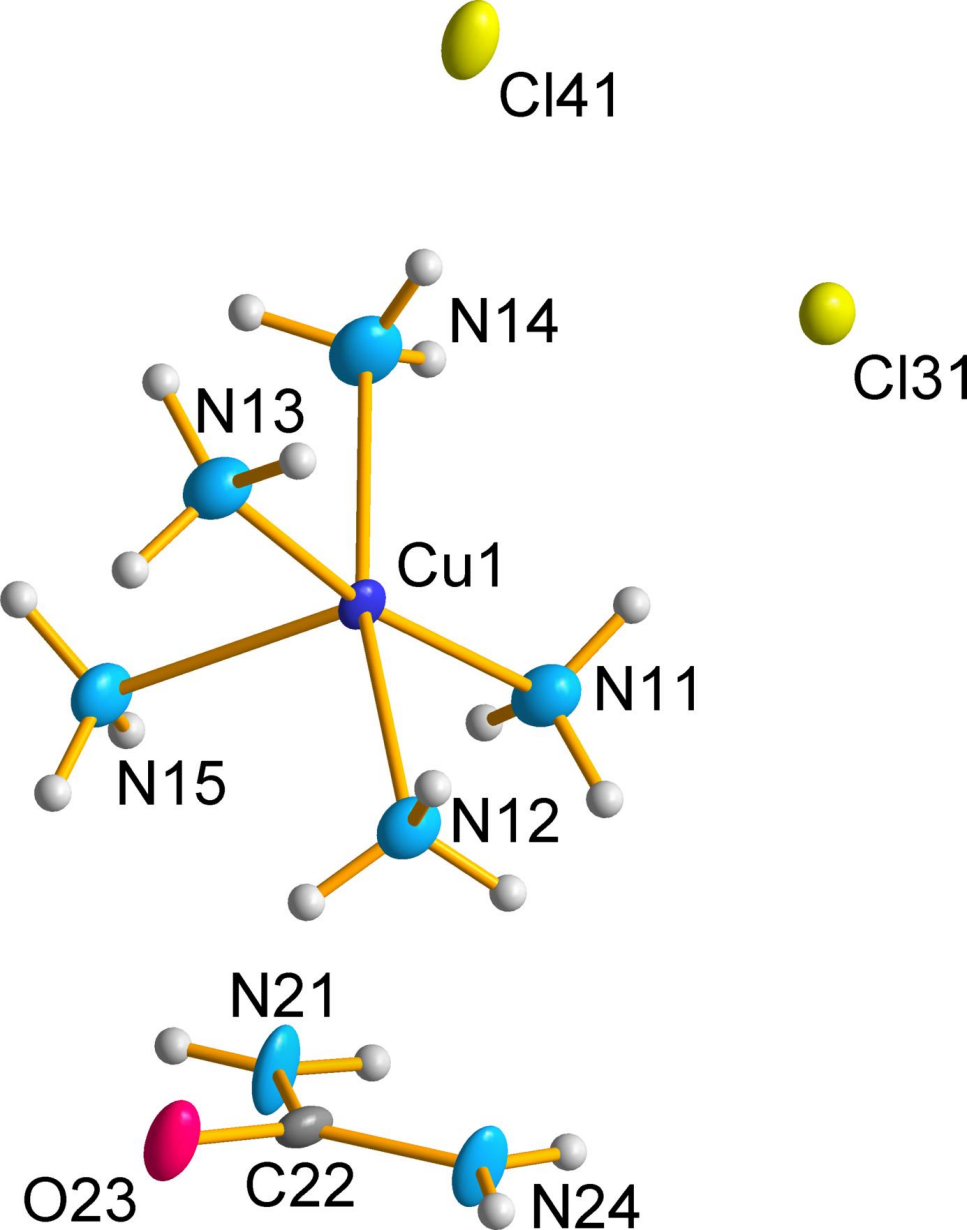
Asymmetric unit of the penta­amine­copper complex showing the atom-labelling scheme. Displacement ellipsoids are drawn at the 75% probability level.

**Figure 2:**
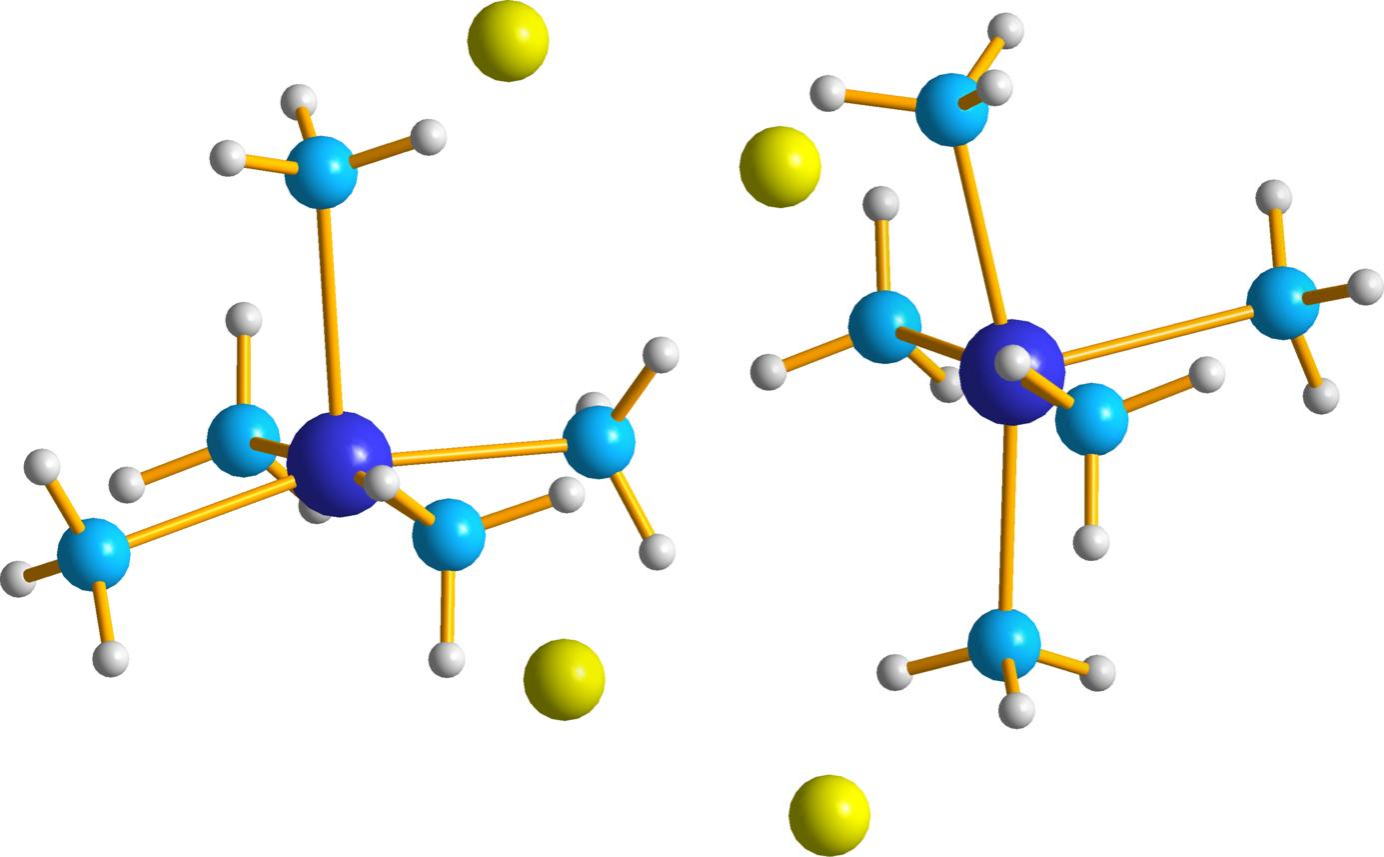
Geometry of the penta­amine­copper(II) complex, with view of the basal site shown confirming the square-based pyramidal geometry

This multi-component crystal (MCC) crystallizes in the *P*2_1_/*n* setting of the space group *P*2_1_/*c* (no. 14). The asymmetric unit (ASU) contains one copper(II) ion coordinated by five ammonia ligands, one urea mol­ecule and two chloride anions (Fig. 1 ▸). The copper ion shows a slightly distorted square-based pyramidal geometry, with four of the ammonia ligands lying on a distorted square plane (N_eq_) and one ammonia forming the vertex (N_ax_). The equatorial Cu—N_eq_ (N11–N14) bond lengths range from 2.0313 (19) to 2.050 (2) Å with an average of 2.039 Å. The axial Cu—N15 bond is longer than the equatorial Cu—N_eq_ (N11–N14) bonds due to anti-bonding electron density down the axial axis (Halcrow, 2013 ▸), with a bond length of 2.2107 (19) Å (Table 1 ▸). The bond angles between the N_eq_—Cu—N_ax_ are slightly above 90° and range from 91.61 (7) to 99.60 (8)° (Table 1 ▸). There are no atoms within the van der Waals radius of the copper at the basal site with the nearest species being the hydrogen atoms on another penta­amine­copper(II) complex (Fig. 2 ▸). This nearest hydrogen atom is located 3.091 Å away, confirming the square-based pyramidal geometry of the complex.

## 3. Supra­molecular features

**Figure 3:**
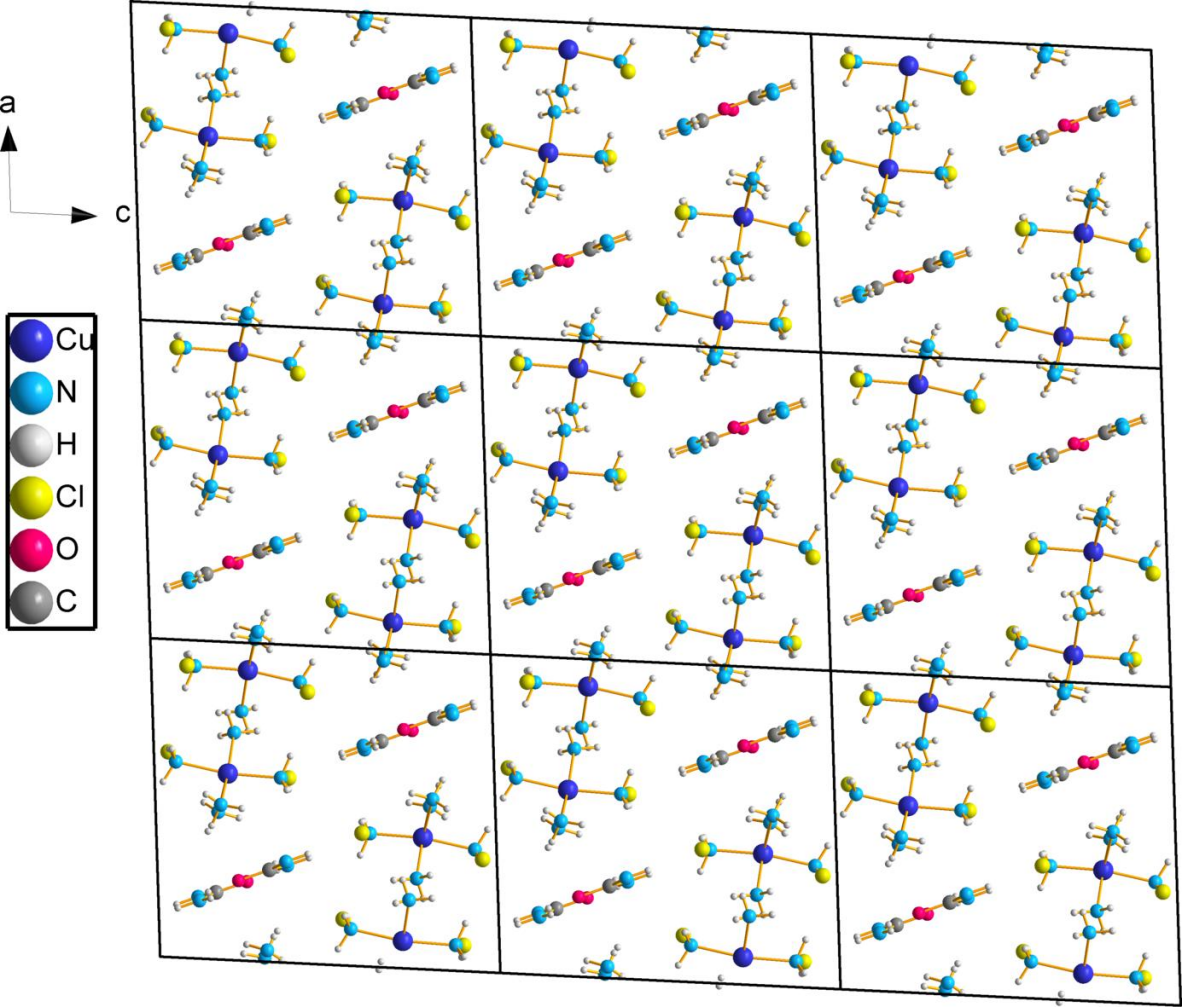
Crystal packing viewed down the *b* axis.

**Figure 4:**
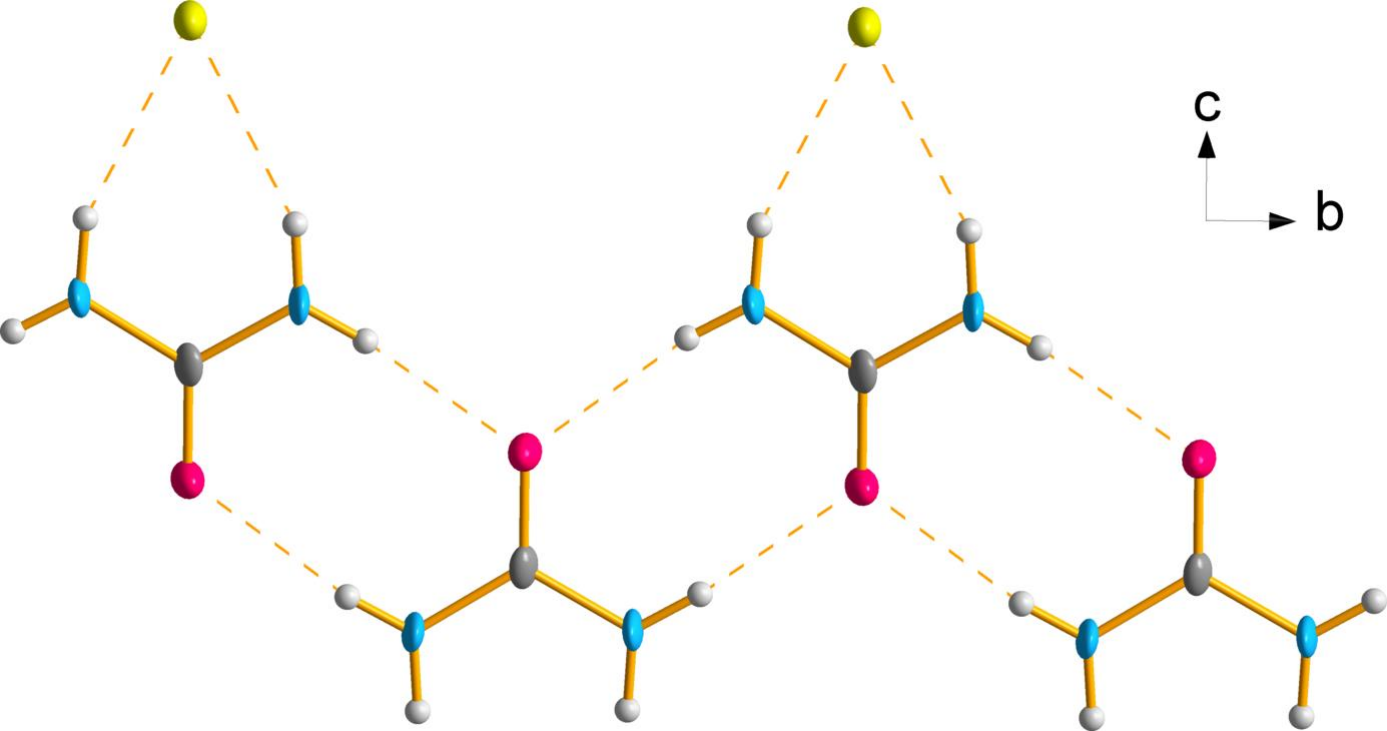
Hydrogen bonding (indicated by the orange dashed lines) between urea mol­ecules and chloride ions viewed down the *a* axis. Displacement ellipsoids are drawn at the 75% probability level.

Each penta­amine­copper(II) complex forms N—H⋯Cl contacts to neighbouring chloride ions. Cl31 forms contacts to four different penta­amine­copper(II) complexes and Cl41 forms contacts to three different penta­amine­copper(II) complexes to create a repeating array of copper ions down the *b* axis (Fig. 3 ▸). These hydrogen bonds have an average N⋯Cl distance of 3.453 Å and range from 3.381 (2) to 3.541 (2) Å (Table 2 ▸). The urea mol­ecules also form N—H⋯Cl41 contacts with an average N⋯Cl41 distance of 3.317 Å (Table 2 ▸). The hydrogens on the urea mol­ecule which do not hydrogen bond with chloride ions instead form hydrogen bonds with other urea mol­ecules with an average N⋯O distance of 2.900 Å (Table 2 ▸). The urea mol­ecules form a ribbon down the *b* axis between copper ions with every other urea mol­ecule facing an alternate direction along the *c* axis (Fig. 4 ▸).

**Figure 5:**
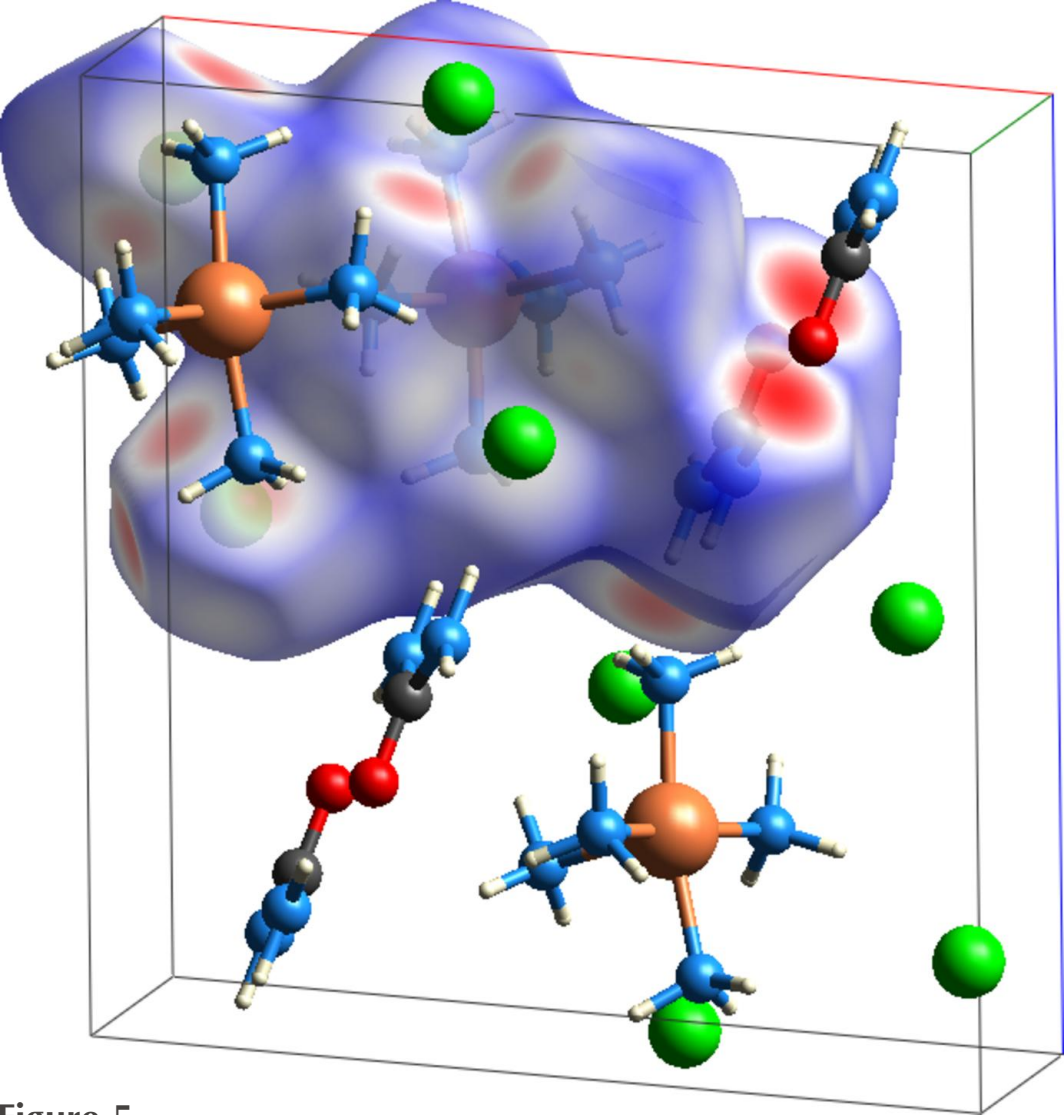
Hirshfeld surface of penta­amine copper complex mapped over *d*_norm_

**Figure 6:**
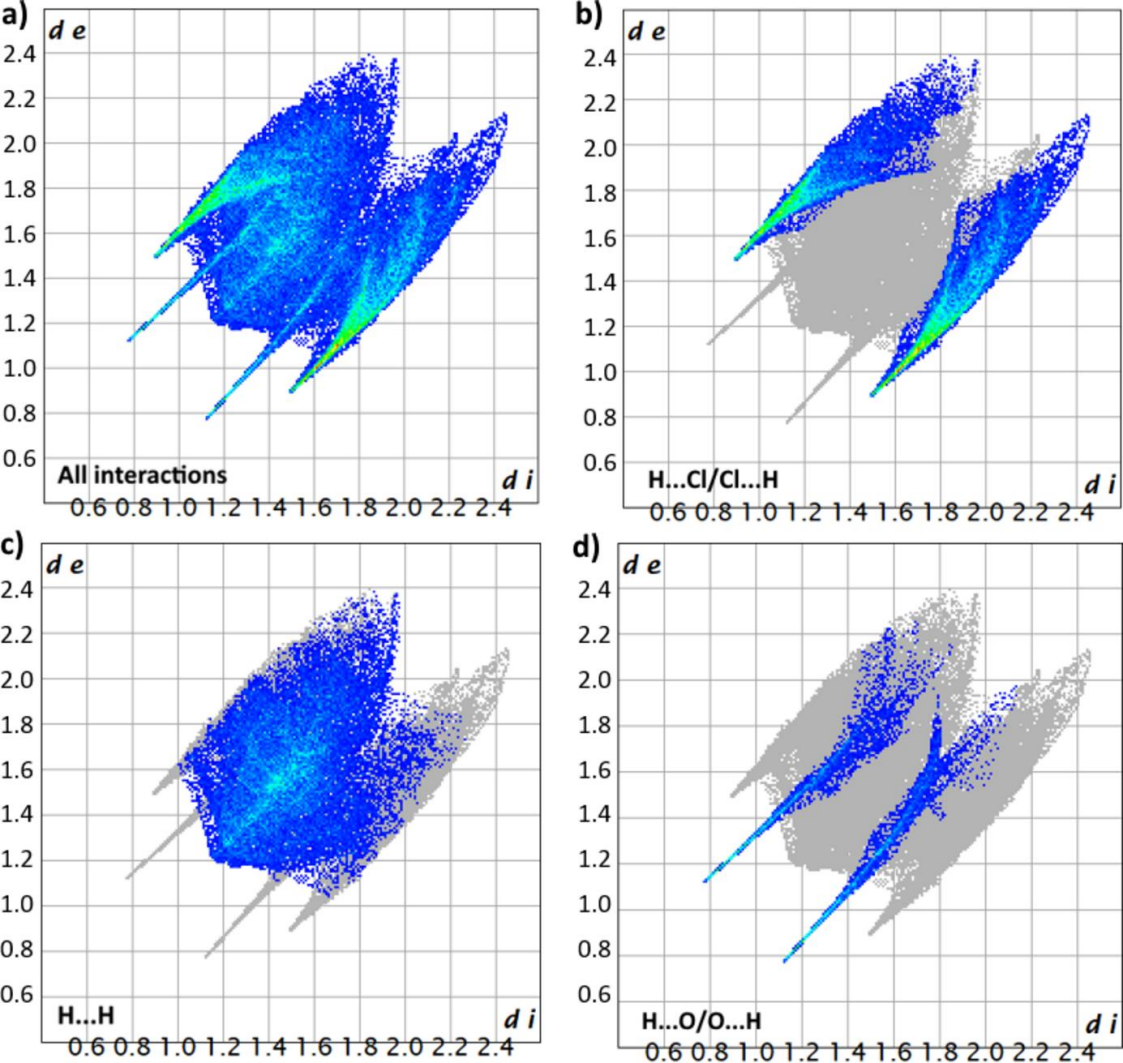
Two-dimensional fingerprint plots showing: (*a*) all inter­actions, (*b*) H⋯Cl/Cl⋯H contacts, (*c*) H⋯H contacts and (*d*) H⋯O/O⋯H contacts.

The Hirshfeld surface analysis (Fig. 5 ▸) and two-dimensional fingerprint plots (Fig. 6 ▸) were calculated using *Crystal Explorer 17* (Spackman *et al.*, 2021 ▸). The colours of the surface relate to the distance of the contacts with red surfaces indicating contacts shorter than the van der Waals radii, white surfaces indicating contacts near the van der Waals radii and blue surfaces indicating contacts longer than the van der Waals radii. N—H⋯O hydrogen bonds between urea mol­ecules, N—H⋯Cl hydrogen bonds between chloride and ammonia, and N—H⋯Cl hydrogen bonds between chloride and urea are indicated by red surfaces. H⋯H contacts (43.1%) and H⋯Cl/Cl⋯H contacts (42.2%) make up the bulk of the contribution to the Hirshfeld surface while H⋯O/O⋯H contacts contribute 9.9%.

## 4. Database survey

There are few penta­amine­copper(II) complexes present in the Cambridge Structural Database (CSD version 5.43, November 2021 update; Groom *et al.*, 2016 ▸) with five reported in total, of which only three displayed square-based pyramidal geometry (refcode: BAWLES, Mironov *et al.*, 2012 ▸; refcode: BAWLOC, Mironov *et al.*, 2012 ▸ and refcode: ONEVIN, Mironov *et al.*, 2011 ▸). Inter­estingly, in one of these entries the MCC contains penta­amine­copper(II) complexes in both square-based pyramidal *and* trigonal–bipyramidal geometry (refcode: BAWLES, Mironov *et al.*, 2012 ▸). These complexes are unusual given that axial bond elongation should result in the axial ligands being more labile. Coupled with ammonia being a volatile ligand this likely gives rise to the rarity of these complexes in the solid state. Due to the paucity of these complexes in the literature, similar complexes were examined where at least three ammonia ligands were present and where one of those was present in the axial position. This only resulted in one additional complex being found, a tri­amine­(ethyl­enedi­amine)­copper(II) complex (refcode: GAFYET, Mironov *et al.*, 2008 ▸). In each of these examples, the copper complex was the cationic counterpart to an anionic cluster. Additionally, in the copper complexes the axial N—Cu bond was always longer than the equatorial N—Cu bond, which is consistent with what has been found in our complex. The literature equatorial N—Cu bonds ranged in value from 1.98 to 2.06 Å with an average of 2.04 Å, while the axial N—Cu bonds were longer and ranged in value from 2.24 to 2.33 Å with an average of 2.28 Å.

## 5. Synthesis and crystallization

Choline chloride (1.3963 g, 10.0 mmol) and urea (1.2058 g, 20.0 mmol) were mixed and heated to 333 K until a homogenous liquid formed. Copper(II) oxalate (0.1682 g, 1.0 mmol) was added to the liquid with stirring to form a suspension. The mixture was poured into a Teflon-lined autoclave and hexa­methyl­ene­tetra­mine (0.0690 g, 0.5 mmol) was added. The autoclave was closed and placed in an oven at 393 K for 48 h before cooling to room temperature over 12 h. The resulting blue liquid was poured into a vial and was capped and was left undisturbed for several months to produce dark-blue needles up to 3 mm in length. Due to the large size of these crystals they were cut to a block shape to be mounted on the diffractometer. Attempts to isolate the crystals were unsuccessful due to the solvent being too viscous to filter and attempts to dilute this with water or alcohol resulted in dissolution of the crystals. A yield of 10 mg was estimated from the crystal size and density.

## 6. Refinement

Crystal data, data collection and structure refinement details are summarised in Table 3 ▸. Urea hydrogen atoms were located in a difference-Fourier map and refined freely. Ammonia hydrogens could be located in a difference-Fourier map but free refinement of them was unstable so they were positioned geometrically and refined as riding with *U*_iso_(H) = 1.2*U*_eq_(N) for NH_3_ hydrogen atoms.

## Supplementary Material

Crystal structure: contains datablock(s) I. DOI: 10.1107/S2056989024004298/oo2003sup1.cif

Structure factors: contains datablock(s) I. DOI: 10.1107/S2056989024004298/oo2003Isup2.hkl

CCDC reference: 2354149

Additional supporting information:  crystallographic information; 3D view; checkCIF report

## Figures and Tables

**Table 1:** Selected geometric parameters (Å, °)

Cu1—N11	2.039 (2)	Cu1—N14	2.0339 (19)
Cu1—N12	2.0313 (19)	Cu1—N15	2.2107 (19)
Cu1—N13	2.050 (2)		
			
N11—Cu1—N15	98.65 (7)	N13—Cu1—N15	91.61 (7)
N12—Cu1—N15	99.60 (8)	N14—Cu1—N15	94.55 (8)

**Table 2:** Hydrogen-bond geometry (Å, °)

*D*—H⋯*A*	*D*—H	H⋯*A*	*D*⋯*A*	*D*—H⋯*A*
N11—H11*A*⋯Cl41^i^	0.91	2.79	3.509 (2)	137
N11—H11*B*⋯Cl31	0.91	2.54	3.443 (2)	170
N11—H11*C*⋯Cl41^ii^	0.91	2.82	3.541 (2)	137
N12—H12*A*⋯Cl31^i^	0.91	2.57	3.461 (2)	167
N12—H12*B*⋯Cl41^i^	0.91	2.67	3.4273 (19)	142
N13—H13*A*⋯Cl31^iii^	0.91	2.49	3.381 (2)	166
N13—H13*C*⋯Cl31^i^	0.91	2.69	3.4752 (15)	145
N14—H14*A*⋯Cl31^ii^	0.91	2.56	3.430 (2)	160
N14—H14*C*⋯Cl41	0.91	2.55	3.418 (2)	159
N15—H15*A*⋯Cl31^ii^	0.91	2.68	3.530 (2)	157
N21—H21*A*⋯O23^iv^	0.79 (3)	2.11 (3)	2.893 (3)	172 (3)
N21—H21*B*⋯Cl41^v^	0.87 (3)	2.50 (3)	3.315 (2)	158 (2)
N24—H24*A*⋯O23^vi^	0.81 (3)	2.10 (3)	2.906 (3)	173 (3)
N24—H24*B*⋯Cl41^v^	0.88 (3)	2.50 (3)	3.319 (2)	157 (3)

Symmetry codes: (i) 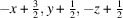 ; (ii) 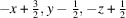 ; (iii) 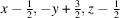 ; (iv) 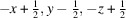 ; (v) 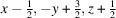 ; (vi) 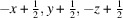 .

**Table 3:** Experimental details

Crystal data	
Chemical formula	[Cu(NH_3_)_5_]Cl_2_·CH_4_N_2_O
*M*_r_	279.67
Crystal system, space group	Monoclinic, *P*2_1_/*n*
Temperature (K)	100
*a*, *b*, *c* (Å)	12.2214 (2), 7.0230 (1), 13.0537 (3)
β (°)	94.511 (2)
*V* (Å^3^)	1116.94 (4)
*Z*	4
Radiation type	Cu *K*α
μ (mm^−1^)	7.01
Crystal size (mm)	0.45 × 0.22 × 0.12

Data collection	
Diffractometer	SuperNova, Dual, Cu at home/near, Atlas
Absorption correction	Multi-scan (*CrysAlis PRO*; Rigaku OD, 2021 ▸)
*T*_min_, *T*_max_	0.229, 1.000
No. of measured, independent and observed [*I* > 2σ(*I*)] reflections	12732, 1977, 1835
*R*_int_	0.059
(sin θ/λ)_max_ (Å^−1^)	0.595

Refinement	
*R*[*F* ^2^ > 2σ(*F* ^2^)], *wR*(*F* ^2^), *S*	0.033, 0.092, 1.08
No. of reflections	1977
No. of parameters	130
H-atom treatment	H atoms treated by a mixture of independent and constrained refinement
Δρ_max_, Δρ_min_ (e Å^−3^)	0.67, −0.80

Computer programs: *CrysAlis PRO* (Rigaku OD, 2021 ▸), *SHELXT2018/2* (Sheldrick, 2015*a* ▸), *SHELXL2018/3* (Sheldrick, 2015*b* ▸) and *OLEX2* (Dolomanov *et al.*, 2009 ▸).

## supplementary crystallographic information

### Crystal data

**Table:**

[Cu(NH_3_)_5_]Cl_2_·CH_4_N_2_O	*F*(000) = 580
*M**_r_* = 279.67	*D*_x_ = 1.663 Mg m^−^^3^
Monoclinic, *P*2_1_/*n*	Cu *K*α radiation, λ = 1.54184 Å
*a* = 12.2214 (2) Å	Cell parameters from 8370 reflections
*b* = 7.0230 (1) Å	θ = 4.8–66.6°
*c* = 13.0537 (3) Å	µ = 7.01 mm^−^^1^
β = 94.511 (2)°	*T* = 100 K
*V* = 1116.94 (4) Å^3^	Block, blue
*Z* = 4	0.45 × 0.22 × 0.12 mm

### Data collection

**Table:**

SuperNova, Dual, Cu at home/near, Atlas diffractometer	1977 independent reflections
Radiation source: micro-focus sealed X-ray tube, SuperNova (Cu) X-ray Source	1835 reflections with *I* > 2σ(*I*)
Mirror monochromator	*R*_int_ = 0.059
Detector resolution: 10.3196 pixels mm^-1^	θ_max_ = 66.6°, θ_min_ = 4.8°
ω scans	*h* = −14→14
Absorption correction: multi-scan (CrysAlisPro; Rigaku OD, 2021)	*k* = −7→8
*T*_min_ = 0.229, *T*_max_ = 1.000	*l* = −15→15
12732 measured reflections	

### Refinement

**Table:**

Refinement on *F*^2^	0 restraints
Least-squares matrix: full	Hydrogen site location: mixed
*R*[*F*^2^ > 2σ(*F*^2^)] = 0.033	H atoms treated by a mixture of independent and constrained refinement
*wR*(*F*^2^) = 0.092	*w* = 1/[σ^2^(*F*_o_^2^) + (0.0515*P*)^2^ + 1.0977*P*] where *P* = (*F*_o_^2^ + 2*F*_c_^2^)/3
*S* = 1.08	(Δ/σ)_max_ = 0.001
1977 reflections	Δρ_max_ = 0.67 e Å^−^^3^
130 parameters	Δρ_min_ = −0.80 e Å^−^^3^

### Special details

**Table:**

Geometry. All esds (except the esd in the dihedral angle between two l.s. planes) are estimated using the full covariance matrix. The cell esds are taken into account individually in the estimation of esds in distances, angles and torsion angles; correlations between esds in cell parameters are only used when they are defined by crystal symmetry. An approximate (isotropic) treatment of cell esds is used for estimating esds involving l.s. planes.

### Fractional atomic coordinates and isotropic or equivalent isotropic displacement parameters (Å^2^)

**Table:**

	*x*	*y*	*z*	*U*_iso_*/*U*_eq_	
Cu1	0.58604 (3)	0.73373 (4)	0.21577 (2)	0.00720 (15)	
N12	0.48687 (15)	0.9643 (3)	0.19244 (13)	0.0121 (4)	
H12A	0.517997	1.048085	0.150183	0.015*	
H12B	0.477733	1.021555	0.253724	0.015*	
H12C	0.420444	0.926610	0.162954	0.015*	
N15	0.46406 (15)	0.5045 (3)	0.18411 (14)	0.0127 (4)	
H15A	0.492053	0.415157	0.142989	0.015*	
H15B	0.401766	0.554421	0.151974	0.015*	
H15C	0.448160	0.449703	0.244330	0.015*	
N11	0.58082 (18)	0.7487 (3)	0.37130 (16)	0.0133 (5)	
H11A	0.543429	0.855049	0.387772	0.016*	
H11B	0.650406	0.754010	0.401582	0.016*	
H11C	0.546287	0.643803	0.393945	0.016*	
N13	0.61575 (18)	0.7501 (2)	0.06366 (15)	0.0137 (5)	
H13A	0.550830	0.753527	0.024449	0.016*	
H13B	0.654939	0.646477	0.046280	0.016*	
H13C	0.654659	0.857730	0.052718	0.016*	
N14	0.71579 (15)	0.5531 (3)	0.23815 (14)	0.0169 (4)	
H14A	0.703349	0.447470	0.198543	0.020*	
H14B	0.724294	0.518588	0.305504	0.020*	
H14C	0.777763	0.612328	0.220417	0.020*	
Cl31	0.85423 (5)	0.74020 (7)	0.45789 (4)	0.01096 (18)	
Cl41	0.91879 (5)	0.74644 (7)	0.10532 (4)	0.01357 (18)	
O23	0.25100 (15)	0.7563 (2)	0.23770 (13)	0.0139 (4)	
N24	0.31771 (16)	0.9190 (3)	0.37916 (15)	0.0131 (4)	
N21	0.30606 (17)	0.5930 (3)	0.38261 (15)	0.0140 (4)	
C22	0.2895 (2)	0.7560 (3)	0.32963 (19)	0.0089 (5)	
H21A	0.290 (2)	0.495 (4)	0.355 (2)	0.011 (7)*	
H24A	0.302 (2)	1.018 (5)	0.350 (2)	0.016 (7)*	
H21B	0.333 (2)	0.601 (4)	0.446 (2)	0.020 (7)*	
H24B	0.346 (2)	0.912 (5)	0.443 (2)	0.026 (8)*	

### Atomic displacement parameters (Å^2^)

**Table:**

	*U*^11^	*U*^22^	*U*^33^	*U*^12^	*U*^13^	*U*^23^
Cu1	0.0095 (2)	0.0032 (2)	0.0089 (2)	0.00087 (11)	0.00069 (14)	0.00008 (10)
N12	0.0148 (9)	0.0080 (10)	0.0138 (8)	0.0009 (8)	0.0024 (7)	0.0003 (7)
N15	0.0150 (9)	0.0066 (10)	0.0167 (9)	−0.0004 (7)	0.0029 (7)	−0.0011 (7)
N11	0.0152 (11)	0.0122 (12)	0.0127 (10)	0.0028 (7)	0.0020 (8)	0.0009 (6)
N13	0.0154 (11)	0.0123 (12)	0.0138 (10)	0.0016 (7)	0.0036 (8)	−0.0002 (7)
N14	0.0189 (9)	0.0114 (11)	0.0201 (9)	0.0050 (8)	−0.0009 (7)	−0.0008 (8)
Cl31	0.0126 (3)	0.0075 (3)	0.0126 (3)	−0.00014 (17)	0.0000 (2)	0.00015 (17)
Cl41	0.0218 (3)	0.0075 (3)	0.0110 (3)	0.00044 (18)	−0.0010 (2)	0.00017 (17)
O23	0.0231 (10)	0.0078 (10)	0.0102 (8)	0.0021 (6)	−0.0026 (7)	−0.0006 (5)
N24	0.0235 (10)	0.0031 (10)	0.0122 (9)	0.0020 (8)	−0.0025 (7)	0.0002 (8)
N21	0.0269 (11)	0.0036 (10)	0.0111 (9)	−0.0021 (8)	−0.0020 (7)	−0.0010 (8)
C22	0.0086 (11)	0.0057 (13)	0.0130 (11)	0.0017 (7)	0.0036 (9)	−0.0008 (7)

### Geometric parameters (Å, º)

**Table:**

Cu1—N11	2.039 (2)	N13—H13A	0.9100
Cu1—N12	2.0313 (19)	N13—H13B	0.9100
Cu1—N13	2.050 (2)	N13—H13C	0.9100
Cu1—N14	2.0339 (19)	N14—H14A	0.9100
Cu1—N15	2.2107 (19)	N14—H14B	0.9100
N12—H12A	0.9100	N14—H14C	0.9100
N12—H12B	0.9100	O23—C22	1.254 (3)
N12—H12C	0.9100	N24—C22	1.346 (3)
N15—H15A	0.9100	N24—H24A	0.81 (3)
N15—H15B	0.9100	N24—H24B	0.87 (3)
N15—H15C	0.9100	N21—C22	1.344 (3)
N11—H11A	0.9100	N21—H21A	0.80 (3)
N11—H11B	0.9100	N21—H21B	0.87 (3)
N11—H11C	0.9100		
			
N11—Cu1—N15	98.65 (7)	H11A—N11—H11B	109.5
N12—Cu1—N15	99.60 (8)	H11A—N11—H11C	109.5
N13—Cu1—N15	91.61 (7)	H11B—N11—H11C	109.5
N14—Cu1—N15	94.55 (8)	Cu1—N13—H13A	109.5
N12—Cu1—N11	92.47 (7)	Cu1—N13—H13B	109.5
N12—Cu1—N13	87.65 (7)	Cu1—N13—H13C	109.5
N12—Cu1—N14	165.49 (8)	H13A—N13—H13B	109.5
N11—Cu1—N13	169.57 (8)	H13A—N13—H13C	109.5
N14—Cu1—N11	88.53 (8)	H13B—N13—H13C	109.5
N14—Cu1—N13	88.78 (8)	Cu1—N14—H14A	109.5
Cu1—N12—H12A	109.5	Cu1—N14—H14B	109.5
Cu1—N12—H12B	109.5	Cu1—N14—H14C	109.5
Cu1—N12—H12C	109.5	H14A—N14—H14B	109.5
H12A—N12—H12B	109.5	H14A—N14—H14C	109.5
H12A—N12—H12C	109.5	H14B—N14—H14C	109.5
H12B—N12—H12C	109.5	C22—N24—H24A	118 (2)
Cu1—N15—H15A	109.5	C22—N24—H24B	118 (2)
Cu1—N15—H15B	109.5	H24A—N24—H24B	124 (3)
Cu1—N15—H15C	109.5	C22—N21—H21A	118.7 (19)
H15A—N15—H15B	109.5	C22—N21—H21B	118 (2)
H15A—N15—H15C	109.5	H21A—N21—H21B	124 (3)
H15B—N15—H15C	109.5	O23—C22—N24	121.39 (19)
Cu1—N11—H11A	109.5	O23—C22—N21	121.60 (19)
Cu1—N11—H11B	109.5	N21—C22—N24	117.0 (2)
Cu1—N11—H11C	109.5		

### Hydrogen-bond geometry (Å, º)

**Table:**

*D*—H···*A*	*D*—H	H···*A*	*D*···*A*	*D*—H···*A*
N11—H11*A*···Cl41^i^	0.91	2.79	3.509 (2)	137
N11—H11*B*···Cl31	0.91	2.54	3.443 (2)	170
N11—H11*C*···Cl41^ii^	0.91	2.82	3.541 (2)	137
N12—H12*A*···Cl31^i^	0.91	2.57	3.461 (2)	167
N12—H12*B*···Cl41^i^	0.91	2.67	3.4273 (19)	142
N13—H13*A*···Cl31^iii^	0.91	2.49	3.381 (2)	166
N13—H13*C*···Cl31^i^	0.91	2.69	3.4752 (15)	145
N14—H14*A*···Cl31^ii^	0.91	2.56	3.430 (2)	160
N14—H14*C*···Cl41	0.91	2.55	3.418 (2)	159
N15—H15*A*···Cl31^ii^	0.91	2.68	3.530 (2)	157
N21—H21*A*···O23^iv^	0.79 (3)	2.11 (3)	2.893 (3)	172 (3)
N21—H21*B*···Cl41^v^	0.87 (3)	2.50 (3)	3.315 (2)	158 (2)
N24—H24*A*···O23^vi^	0.81 (3)	2.10 (3)	2.906 (3)	173 (3)
N24—H24*B*···Cl41^v^	0.88 (3)	2.50 (3)	3.319 (2)	157 (3)

Symmetry codes: (i) −*x*+3/2, *y*+1/2, −*z*+1/2; (ii) −*x*+3/2, *y*−1/2, −*z*+1/2; (iii) *x*−1/2, −*y*+3/2, *z*−1/2; (iv) −*x*+1/2, *y*−1/2, −*z*+1/2; (v) *x*−1/2, −*y*+3/2, *z*+1/2; (vi) −*x*+1/2, *y*+1/2, −*z*+1/2.
